# Increased circulating myeloid-derived suppressor cells in vivax malaria and severe falciparum malaria

**DOI:** 10.1186/s12936-022-04268-6

**Published:** 2022-09-06

**Authors:** Leo Leonardo, Enny Kenangalem, Jeanne R. Poespoprodjo, Rintis Noviyanti, Ric N. Price, Nicholas M. Anstey, Gabriela Minigo, Steven Kho

**Affiliations:** 1 Papuan Health and Community Development Foundation, Timika, Papua Indonesia; 2 Department of Pediatrics, University of Gadjah Mada, Yogyakarta, Indonesia; 3 Eijkman Institute for Molecular Biology, Jakarta, Indonesia; 4 Menzies School of Health Research and Charles Darwin University, Darwin, NT Australia; 5 Centre for Tropical Medicine, Nuffield Department of Clinical Medicine, University of Oxford, Oxford, OX37LJ UK; 6 Mahidol-Oxford Tropical Medicine Research Unit (MORU), Faculty of Tropical Medicine, Mahidol University, Bangkok, Thailand; 7 College of Health and Human Sciences, Charles Darwin University, Darwin, NT Australia

**Keywords:** MDSC, Malaria, Vivax, Severe falciparum, Clinical

## Abstract

**Background:**

Circulating myeloid-derived-suppressor-cells (MDSC) with immunosuppressive function are increased in human experimental *Plasmodium falciparum* infection, but have not been studied in clinical malaria.

**Methods:**

Using flow-cytometry, circulating polymorphonuclear-MDSC were evaluated in cryopreserved samples from patients with uncomplicated *Plasmodium vivax* (n = 8) and uncomplicated (n = 4) and severe (n = 16) falciparum malaria from Papua, Indonesia.

**Results:**

The absolute number of circulating polymorphonuclear-MDSC were significantly elevated in severe falciparum malaria patients compared to controls (n = 10). Polymorphonuclear-MDSC levels in uncomplicated vivax malaria were also elevated to levels comparable to that seen in severe falciparum malaria.

**Conclusion:**

Control of expansion of immunosuppressive MDSC may be important for development of effective immune responses in falciparum and vivax malaria.

## Background

Malaria remains a major life-threatening infectious disease in tropical and subtropical countries. The World Health Organization reported an increase in malaria cases and deaths in 2020, with an estimated 241 million malaria cases and 627,000 deaths worldwide [1]. *Plasmodium falciparum* is responsible for over 97% of global malaria cases and nearly all malaria deaths [1]. Outside of sub-Saharan Africa *Plasmodium vivax* also causes significant morbidity [1]. Failure to control the expansion of *P. falciparum* parasite biomass, including delayed treatment [2], can result in progression to severe malaria and death [3]. The way in which the immune system responds to *Plasmodium* infection remains incompletely understood, although failure to control *Plasmodium* infection and clinical disease are associated with an immunoregulatory immune response [4, 5].

Myeloid-derived-suppressor-cells (MDSC) are a heterogenous group of polymorphonuclear-type (PMN-MDSC) or monocytic-type MDSC with strong immunosuppressive activity against T-cells, that can regulate the function of other immune cells. MDSC are emerging as key pathological mediators of disease including in cancer [6], sepsis [7, 8] and SARS-CoV-2 [9, 10], and also play a role in physiological maternal-fetal tolerance [11, 12]. PMN-MDSC are recognized as pathologically activated neutrophils and display distinct pro-inflammatory and immunosuppressive gene signatures compared to classical neutrophils [13, 14]. Recent work in controlled human malaria infections (CHMI) with *P. falciparum* sporozoites report expansion of PMN-MDSC in individuals developing blood-stage parasitaemia with suppression of CD4^+^ and CD8^+^ T cell proliferation [15]. MDSC expansion is also reported in murine models of malaria [16–18]. Despite their important immunoregulatory roles, MDSC have not been studied in patients with clinical malaria.

During peripheral blood separation by density gradient centrifugation, low-density PMN-MDSC co-purify with peripheral blood mononuclear cells (PBMC) [7], enabling their separation from high-density classical neutrophils sharing the same surface markers [6]. To determine whether PMN-MDSC are elevated in clinical malaria, we evaluated PMN-MDSC in cryopreserved PBMC samples from hospitalised patients with clinical *P. falciparum* and *P. vivax* malaria, including patients with severe disease, and compared their levels to uninfected individuals residing in the same endemic area.

## Methods

### Study site and samples

In the eastern Indonesian province of Papua the prevalence of *P. falciparum* and *P. vivax* are similar [19], with acute uncomplicated and severe malaria occurring in both children and adults [20]. In clinical studies conducted in Timika between 2011 and 2013 [21], peripheral venous blood samples were collected from malaria patients infected with *P. falciparum* or *P. vivax* attending Rumah Sakit Mitra Masyarakat Hospital, and had PBMC cryopreserved. Definitions for enrolment of uncomplicated and severe malaria cases (modified 2000 WHO research criteria; Table 2) are described previously [22]. PBMC from Timika household survey participants collected in the same period who were polymerase-chain-reaction-negative for *Plasmodium* and had no history of fever in the preceding month were included as controls [23]. Participant demographic and clinical data were recorded on standardized forms. Differential full blood counts were collected on an automated analyser (Sysmex, Illinois, US). Giemsa-stained blood smears were read by experienced research microscopists to determine parasitaemia.

### Flow cytometry

To examine PMN-MDSC, two-hundred thousand PBMC were stained with antibodies, comprising anti-CD66b (clone G10F5) conjugated to fluorescein isothiocyanate, anti-CD14 (clone MφP9) conjugated to allophycocyanin (both from BD Biosciences, San Jose, CA), CD11b (clone LM2) conjugated to phycoerythrin and anti-CD15 (clone W6D3) conjugated to peridinin chlorophyll protein complex (PerCP) (both from Biolegend, San Diego, CA). To provide insight into immunosuppressive function, we stained one million PBMC with anti-CD4 (clone RPA-T4) conjugated to PerCP to count CD4+ T cells in the same samples. Stained cells were acquired on a portable BD Accuri C6 flow cytometer (BD Biosciences).

### Data analysis

Flow cytometric data were analysed on FlowJo v10 (TreeStar, Ashland, OR) and statistical analysis was conducted using Graphpad Prism v9 (La Jolla, CA). PMN-MDSC levels were reported as absolute numbers per microlitre of blood, calculated from the number of PMN-MDSC events relative to events of mononuclear cells for which automated cell counts were available. More specifically, the number of PMN-MDSC events was divided by the number of PBMC events (gated on lymphocytes and monocytes) and multiplied by the absolute number of PBMC (sum of automated lymphocyte and monocyte counts). The Kruskall–Wallis test with Dunn’s multiple comparison was used for comparison of continuous variables between groups. The chi-square test was used for comparison of categorical variables. The Mann–Whitney test was used for comparisons of severity criteria in severe malaria cases. Correlations were evaluated using the Spearman test (severe falciparum and uncomplicated vivax malaria were analysed as separate groups).

### Ethics

The work was approved by the Human Research Ethics Committees of Gadjah Mada University (KE/FK/763/EC and KE/FK/544/EC) and Menzies School of Health Research (HREC 10-1397 and 10-1434). Written informed consent was obtained from all participants.

## Results

### Study participants

PBMC preparations from a total of 38 individuals were examined, of whom 8 had uncomplicated *P. vivax*, 20 had *P. falciparum* infection (4 uncomplicated and 16 severe) and 10 were PCR-negative healthy controls (Table 1). All participants were adults (> 18 years) except for three patients with *P. falciparum* infection who were aged 7, 9 and 13 years. There was an underrepresentation of Papuans in the control group; however, all were residents of Timika for at least 2 years prior to enrolment [23]. Neutrophil counts were higher in uncomplicated vivax and severe falciparum malaria relative to controls, and haemoglobin levels were reduced in severe falciparum malaria (Table 1). In severe malaria, 50% of adults (7/14) had neutrophilia (> 7400 neutrophils per microlitre of blood; Timika adult population mean plus two standard deviations, n = 794 household survey). The most common criteria for disease severity were cerebral malaria and hyperparasitaemia (Table 2).

**Table 1:** Participant baseline characteristics

	Controls	Uncomplicated malaria		Severe falciparum malaria	
		*P. vivax*	*P. falciparum*		
Sample size (n)	10	8	4	16	
Gender (n of male [%])	3 (30)	6 (75)	1 (25)	9 (56)	
Age (median [range])	33 (19–60)	28 (18–54)	21 (13–40)	25 (7–61)	
Children, < 15 years (n [%])	0	0	1 (25)	2 (13)	
Ethnicity (n of Papuan [%])	1 (10)	3 (38)	4 (100)**	13 (81)***	
Parasitaemia, count/µL (median [range])	0	5400 (2200–20,100)	10,400 (3200–12,700)	35,200 (75–1,800,000)	
Ha4emoglobin, g/dL (median [range])	13.5 (10.6–16.2)	14.3 (10.7–16.3)	13.3 (10.8–14.2)	11.1 (7.3–14.1)*	
White cells ×10^3^/µL (median [range])	7.1 (5.4–12.0)	7.6 (4.2–8.7)	7.0 (4.2–8.3)	10.1 (5.6–50.8)	
Neutrophils ×10^3^/µL (median [range])	3.2 (2.6–4.0)	5.4 (4.3–6.4)*	5.5 (2.5–6.4)	6.7 (4.8–11.8)****	
Neutrophilia in adults (n [%])	0	0	0	7 (50)*	
Monocytes ×10^3^/µL (median [range])	0.5 (0.4–0.8)	0.7 (0.2–1.8)	0.7 (0.3–1.1)	0.8 (0.7–2.1)*	
Lymphocytes ×10^3^/µL (median [range])	2.6 (2.4–2.9)	0.8 (0.5–1.1)***	1.0 (0.9–1.3)*	1.6 (1.1–3.2)	
CD4+ T-cells per µL (median [range])	1060 (730–1470)	260 (40–980)**	980 (370–990)	440 (50–1540)**	

T cell data not available for 1 patient in each group (2 in severe)

Laboratory data (except parasitaemia) missing for 1 severe patient

Criteria for neutrophilia comprised neutrophil counts greater than 7400 per microliter blood (the Timika adult population mean plus two standard deviations, n = 794 household survey)

Categorical variables are compared using the Chi-square test and continuous variables using the Kruskal–Wallis test with Dunn’s multiple comparison (significantly different to controls, *p < 0.05, **p < 0.005, ***p < 0.0005, ****p < 0.0001)

**Table 2:** Manifestations of severe malaria in 16 severe falciparum malaria cases

Manifestations	n of patients with single manifestation	Patients with > 1 manifestation				
		Patient A	Patient B	Patient C	Patient D	Patient E
Cerebral malaria^a^	5					
Jaundice^b^	1		x		x	x
Acute renal failure^c^	1	x	x			x
Hyperparasitaemia^d^	3			x	x	
Respiratory distress^e^	0	x		x		
Prostration^f^	1			x		

^a^Glasgow coma score ≤ 10 for > 30 min

^b^Visible jaundice and either > 100,000 parasites/µL or creatinine > 1.5 mg/dL

^c^Creatinine > 3 mg/dL with or without urine output < 400mL/day

^d^Asexual parasitaemia > 10%

^e^Respiratory rate > 32/min or low oxygen saturation (< 94%)

^f^Unable to sit unaided

### PMN-MDSC expansion in acute uncomplicated and severe malaria

**Fig. 1:**
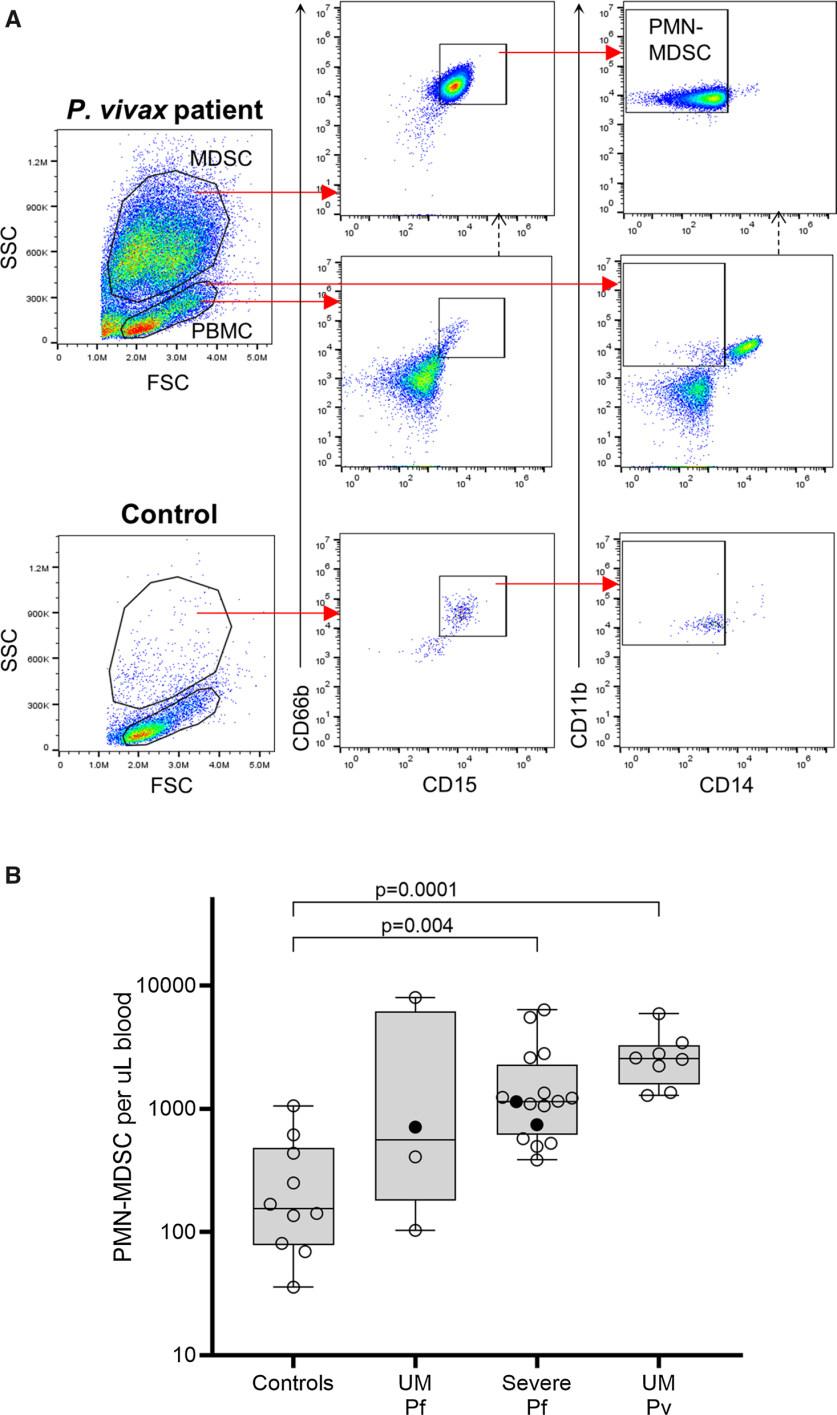
PMN-MDSC phenotype and absolute numbers in clinical malaria. PMN-MDSC were identified by 4-color flow cytometry as CD15^+^CD66b^+^CD11b^+^CD14^−^ cells in PBMC samples from 10 controls, 12 UM patients (4 Pf and 8 Pv) and 16 cases of severe Pf malaria. Representative gating strategy from a Pv patient and control is shown in panel **A**. The absolute number of circulating PMN-MDSC were compared between malaria patients and controls using the Kruskal–Wallis test with Dunn’s multiple comparison (**B**). Plots show individual datapoints with median, interquartile-range and range. Bold data points are children < 15 years. PMN-MDSC: polymorphonuclear-type myeloid-derived suppressor cells; UM: uncomplicated malaria; Pf: *P. falciparum*; Pv: *P. vivax*

**Fig. 2:**
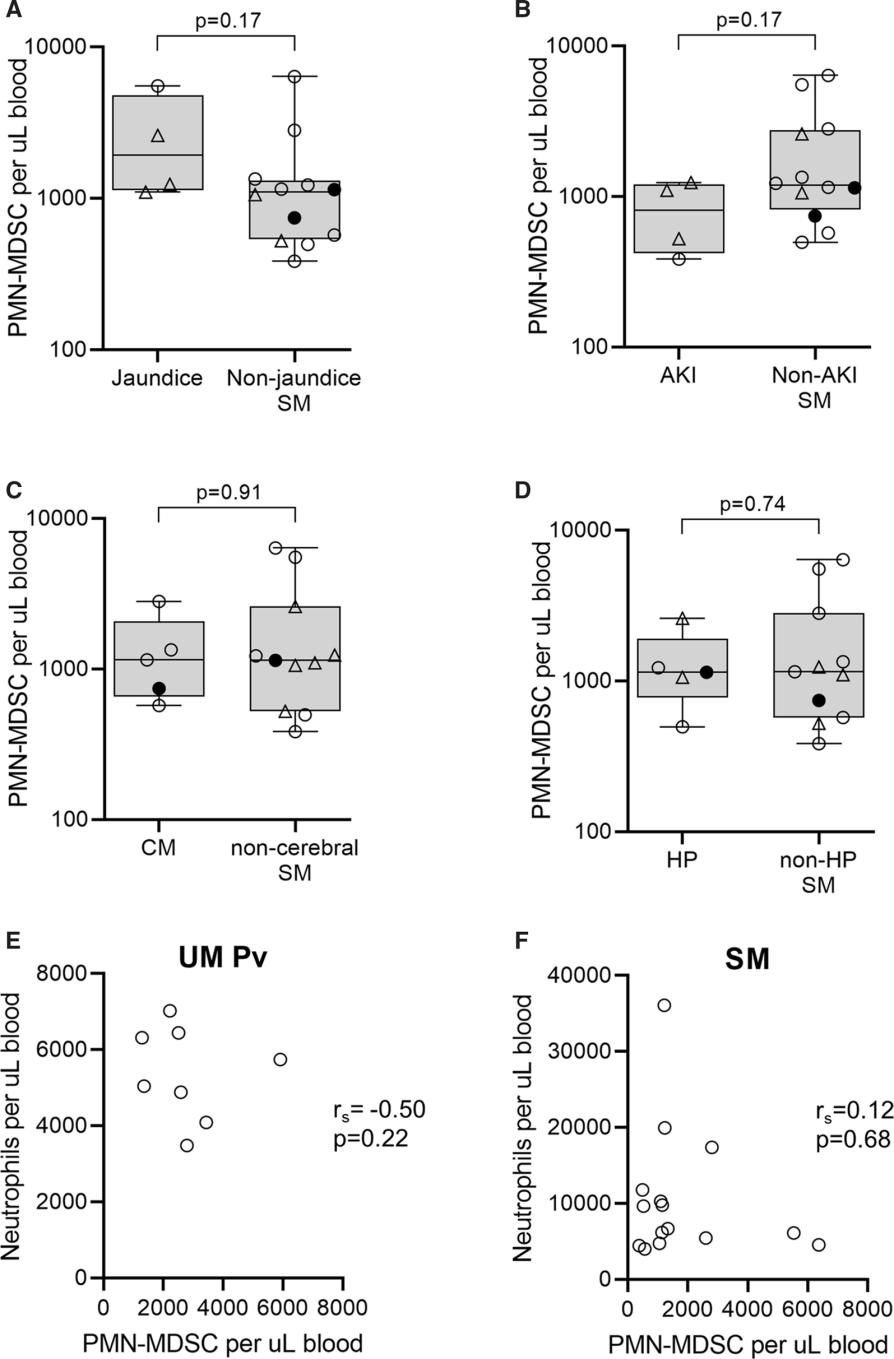
MDSC in severe malaria and correlations with automated neutrophil counts. The absolute number of circulating PMN-MDSC were compared between severe malaria patients with and without jaundice (**A**), AKI (**B**), CM (**C**) and HP (**D**). Plots show individual datapoints with median, interquartile-range and range. Bold data points are children < 15 years and triangular data points are patients with more than 1 severity criteria. The Mann–Whitney test was used to compare groups. The associations between circulating PMN-MDSC numbers and automated neutrophils counts were determined in patients with uncomplicated Pv (**E**) and severe malaria (**F**). PMN-MDSC: polymorphonuclear-type myeloid-derived suppressor cells; SM: severe malaria; AKI: acute kidney injury; CM: cerebral malaria; HP: hyperparasitaemia; UM: uncomplicated malaria; Pv: *P. vivax*

Circulating PMN-MDSC were phenotyped by flow cytometry based on side-scatter (SCC) properties and cell surface markers. After gating on SCC^hi^, PMN-MDSC were identified as CD15^+^CD66b^+^CD11b^+^CD14^−^ cells (Fig. 1A) [6]. The absolute number of circulating PMN-MDSC were increased in all clinical malaria patients, significant in severe falciparum (median 1150 [range: 384–6370] per µL blood, p = 0.004) and uncomplicated vivax malaria (median 2550 [range: 1290–5910] per µL blood, p < 0.0001) relative to controls (median 155 [range: 36–1060] per µL blood, Fig. 1B). PMN-MDSC levels in uncomplicated vivax malaria were comparably elevated to levels seen in severe falciparum malaria. The expansion of circulating PMN-MDSC seen in the 4 cases of uncomplicated falciparum malaria did not reach statistical significance (median 559 [range: 103–8020] per µL blood, p = 0.47). PMN-MDSC levels remained significantly greater in severe falciparum malaria after excluding children (p = 0.005). Among the different severity criteria in patients with severe disease, those with jaundice displayed a trend towards higher circulating PMN-MDSC levels compared to those without jaundice (Fig. 2A) and those with acute kidney injury (AKI) had reduced PMN-MDSC levels (Fig. 2B); the analyses were likely underpowered with neither comparisons reaching statistical significance. Patients with cerebral malaria or hyperparasitaemia displayed no obvious differences in PMN-MDSC levels compared to patients with other identified severity criteria (Fig. 2C, D).

### PMN-MDSC relationships with neutrophils, CD4+ T cells, parasitaemia and age

Whether circulating PMN-MDSC levels were associated with automated neutrophil counts was assessed. Despite sharing surface markers and ancestry [6], there was no significant correlations between MDSC and neutrophils in uncomplicated vivax malaria (r_s_= − 0.50, p = 0.22; Fig. 2E) or severe falciparum malaria (r_s _= 0.12, p = 0.68; Fig. 2F). Relationships with CD4^+^ T cells and lymphocyte counts were examined to gain insight into MDSC immunosuppressive function. In accordance with previous reports from the same study area [23], loss of circulating CD4+ T cells in uncomplicated vivax and severe falciparum malaria relative to controls was observed (Table 1) but this was not correlated with changes in PMN-MDSC in either patient group (r_s_ = 0.21, p = 0.66 and r_s_ = 0.20, p = 0.48, respectively). Further, PMN-MDSC levels were not correlated with automated lymphocyte counts for both species (r_s_ = 0.36, p = 0.39 and r_s_ = 0.31, p = 0.27, respectively). Lastly, it was determined if circulating PMN-MDSC levels were associated with parasitaemia or patient age. While no relationships were observed with parasitaemia (r_s _= 0.31, p = 0.46 in uncomplicated vivax and r_s_ = − 0.22, p = 0.40 in severe falciparum malaria), an inverse association with age was observed in *P. vivax* infection (r_s_ = − 0.69, p = 0.07).

## Discussion

This is the first study evaluating MDSC in clinical malaria. Patients with uncomplicated *P. vivax*, *P. falciparum* and severe falciparum malaria display increased PMN-MDSC in circulating blood. Circulating PMN-MDSC were at comparably high levels in uncomplicated *P. vivax* and severe falciparum malaria. PMN-MDSC may contribute to the greater immunoregulatory response previously observed in *P. vivax* infections [4]. Severe falciparum malaria was not associated with higher levels of PMN-MDSC compared to uncomplicated disease, though the power to test this was limited. The expansion of circulating PMN-MDSC seen in clinical *P. falciparum* infections is consistent with elevated PMN-MDSC reported in CHMI developing *P. falciparum* parasitaemia [15]. With their known immunosuppressive function, our findings suggest that PMN-MDSC are important immune regulators in clinical disease and may contribute to malaria-induced immunosuppression.

PMN-MDSC mediate T cell dysfunction and have been shown to suppress ex-vivo T cell proliferation in CHMI volunteers [15]. In the present cohort, loss of circulating CD4^+^ T cells was not associated with PMN-MDSC levels in peripheral blood, nor were there any correlations between PMN-MDSC and lymphocyte counts. Larger studies directly assessing the immunosuppressive activity of circulating PMN-MDSC in clinical malaria are warranted. Analysis of severe falciparum malaria as a single group indicated similar PMN-MDSC levels to uncomplicated malaria, though the sample size was underpowered to confirm this observation. The greater expansion of PMN-MDSC in severely ill patients with jaundice compared to those without was not statistically significant but is plausible, and is consistent with a previous study reporting increased PMN-MDSC in cancer patients with related jaundice [24]. Conversely, the trend towards reduced circulating PMN-MDSC in AKI compared to other severity criteria is in-line with cells of this phenotype being recruited to the site of injury in mice models [25], potentially minimising the numbers remaining in peripheral blood. Migration of circulating MDSC to the tissues is also reported in experimental murine cerebral malaria [26]. However, in the present cohort, the similar circulating PMN-MDSC levels seen in cerebral and non-cerebral severe malaria did not reflect this.

PMN-MDSC are pathologically activated neutrophils distinguished from classical neutrophils by density gradient separation [6]. In the present study, the absence of correlations between circulating PMN-MDSC and automated neutrophil counts in uncomplicated vivax and severe falciparum malaria further confirmed that our identification of PMN-MDSC from co-purification with low-density PBMC were unlikely to be contaminated with high-density classical neutrophils, and that PMN-MDSC expansion was not related to the neutrophilia associated with severe falciparum malaria seen here and elsewhere [27]. Inflammatory factors and parasite-derived molecules have been shown to trigger MDSC development in parasitic infections [16]. Furthermore, murine malaria studies suggest that MDSC can contribute directly to parasite clearance and prevent pathology [18]. In patients with malaria, no relationship between circulating PMN-MDSC and parasitaemia was observed, suggesting lack of MDSC anti-parasitic activity or direct parasite-driven effects on MDSC expansion. It was not feasible to evaluate soluble measures of inflammation or more accurate measures of parasite biomass to assess these relationships further. Patient age was associated inversely with the number of circulating PMN-MDSC in uncomplicated vivax malaria, in contrast to the age-related increase in PMN-MDSC frequency that is known to occur physiologically in humans [28]. It is speculated that chronic exposure to *P. vivax* may be affecting the age-related alterations in MDSC homeostasis, though the data supporting this should be interpreted with caution given the small sample size in our cohort.

## Conclusion

Circulating PMN-MDSC expand in clinical vivax and severe falciparum malaria, and may contribute to the immunosuppression previously observed in clinical disease from both species. In uncomplicated *P. vivax* infections, PMN-MDSC concentrations were influenced by age and were at least as high as levels in severe *P. falciparum* infections. In severe disease, the level of PMN-MDSC expansion may contribute to different severe manifestations. Larger studies on the kinetics of PMN-MDSC (as well as monocytic-MDSC) in clinical disease and their role in asymptomatic infections are warranted to evaluate MDSC as a potential control target to improve immune responsiveness for both species.

## Declarations

### Availability of data and materials

All data are available from the authors upon reasonable request. Biological materials are no longer available.

## References

[1] WHO (2021). World malaria report 2021.

[2] Mousa A, Al-Taiar A, Anstey NM, Badaut C, Barber BE, Bassat Q (2020). The impact of delayed treatment of uncomplicated *P. falciparum* malaria on progression to severe malaria: a systematic review and a pooled multicentre individual-patient meta-analysis. PLoS Med.

[3] WHO (2014). Severe malaria. Trop Med Int Health.

[4] Woodberry T, Loughland JR, Minigo G, Burel JG, Amante FH, Piera KA (2017). Early immune regulatory changes in a primary controlled human *Plasmodium vivax* infection: cd1c + myeloid dendritic cell maturation arrest, induction of the kynurenine pathway, and regulatory T cell activation. Infect Immun.

[5] Minigo G, Woodberry T, Piera KA, Salwati E, Tjitra E, Kenangalem E (2009). Parasite-dependent expansion of TNF receptor II–positive regulatory T cells with enhanced suppressive activity in adults with severe malaria. PLoS Pathog.

[6] Veglia F, Sanseviero E, Gabrilovich DI (2021). Myeloid-derived suppressor cells in the era of increasing myeloid cell diversity. Nat Rev Immunol.

[7] Darcy CJ, Minigo G, Piera KA, Davis JS, McNeil YR, Chen Y (2014). Neutrophils with myeloid derived suppressor function deplete arginine and constrain T cell function in septic shock patients. Crit Care.

[8] Mathias B, Delmas AL, Ozrazgat-Baslanti T, Vanzant EL, Szpila BE, Mohr AM (2017). Human myeloid-derived suppressor cells are associated with chronic immune suppression after severe sepsis/septic shock. Ann Surg.

[9] Agrati C, Sacchi A, Bordoni V, Cimini E, Notari S, Grassi G (2020). Expansion of myeloid-derived suppressor cells in patients with severe coronavirus disease (COVID-19). Cell Death Differ.

[10] Sacchi A, Grassi G, Bordoni V, Lorenzini P, Cimini E, Casetti R (2020). Early expansion of myeloid-derived suppressor cells inhibits SARS-CoV-2 specific T-cell response and may predict fatal COVID-19 outcome. Cell Death Dis.

[11] Ostrand-Rosenberg S, Sinha P, Figley C, Long R, Park D, Carter D (2017). Frontline science: myeloid-derived suppressor cells (MDSCs) facilitate maternal–fetal tolerance in mice. J Leukoc Biol.

[12] Zhao A-M, Xu H-J, Kang X-M, Zhao A-M, Lu L-M (2016). New insights into myeloid-derived suppressor cells and their roles in feto-maternal immune cross-talk. J Reprod Immunol.

[13] Fridlender ZG, Sun J, Mishalian I, Singhal S, Cheng G, Kapoor V (2012). Transcriptomic analysis comparing tumor-associated neutrophils with granulocytic myeloid-derived suppressor cells and normal neutrophils. PLoS ONE.

[14] Condamine T, Dominguez GA, Youn J-I, Kossenkov AV, Mony S, Alicea-Torres K (2016). Lectin-type oxidized LDL receptor-1 distinguishes population of human polymorphonuclear myeloid-derived suppressor cells in cancer patients. Science Immunol.

[15] Calle CL, Fendel R, Singh A, Richie TL, Hoffman SL, Kremsner PG (2021). Expansion of functional myeloid-derived suppressor cells in controlled human malaria infection. Front Immunol.

[16] Van Ginderachter JA, Beschin A, De Baetselier P, Raes G (2010). Myeloid-derived suppressor cells in parasitic infections. Eur J Immunol.

[17] Sponaas A-M, Freitas do Rosario AP, Voisine C, Mastelic B, Thompson J, Koernig S (2009). Migrating monocytes recruited to the spleen play an important role in control of blood stage malaria. Blood.

[18] Belyaev NN, Brown DE, Diaz A-IG, Rae A, Jarra W, Thompson J (2010). Induction of an IL7-R + c-Kithi myelolymphoid progenitor critically dependent on IFN-γ signaling during acute malaria. Nat Immunol.

[19] Pava Z, Burdam FH, Handayuni I, Trianty L, Utami RAS, Tirta YK (2016). Submicroscopic and asymptomatic *Plasmodium* parasitaemia associated with significant risk of anaemia in Papua, Indonesia. PLoS ONE.

[20] Karyana M, Burdarm L, Yeung S, Kenangalem E, Wariker N, Maristela R (2008). Malaria morbidity in Papua Indonesia, an area with multidrug resistant *Plasmodium vivax* and *Plasmodium falciparum*. Malar J.

[21] Rambhatla JS, Tonkin-Hill GQ, Takashima E, Tsuboi T, Noviyanti R, Trianty L (2021). Identifying targets of protective antibodies against severe malaria in Papua, Indonesia using locally expressed domains of *Plasmodium falciparum* erythrocyte membrane protein 1. Infect Immun.

[22] Yeo TW, Lampah DA, Gitawati R, Tjitra E, Kenangalem E, McNeil YR (2007). Impaired nitric oxide bioavailability and l-arginine–reversible endothelial dysfunction in adults with falciparum malaria. J Exp Med.

[23] Kho S, Marfurt J, Noviyanti R, Kusuma A, Piera KA, Burdam FH (2015). Preserved dendritic cell HLA-DR expression and reduced regulatory T cell activation in asymptomatic *Plasmodium falciparum* and *P. vivax* infection. Infect Immun.

[24] Zhang J, Xu X, Shi M, Chen Y, Yu D, Zhao C (2017). CD13hi neutrophil-like myeloid-derived suppressor cells exert immune suppression through Arginase 1 expression in pancreatic ductal adenocarcinoma. Oncoimmunology.

[25] Zhang C, Wang S, Li J, Zhang W, Zheng L, Yang C (2017). The mTOR signal regulates myeloid-derived suppressor cells differentiation and immunosuppressive function in acute kidney injury. Cell Death Dis.

[26] Schumak B, Klocke K, Kuepper JM, Biswas A, Djie-Maletz A, Limmer A (2015). Specific depletion of Ly6Chi inflammatory monocytes prevents immunopathology in experimental cerebral malaria. PLoS ONE.

[27] Kho S, Minigo G, Andries B, Leonardo L, Prayoga P, Poespoprodjo JR (2019). Circulating neutrophil extracellular traps and neutrophil activation are increased in proportion to disease severity in human malaria. J Infect Dis.

[28] Verschoor CP, Johnstone J, Millar J, Dorrington MG, Habibagahi M, Lelic A (2013). Blood CD33(+)HLA-DR(–) myeloid-derived suppressor cells are increased with age and a history of cancer. J Leukoc Biol.

